# Frequency of tuberculosis at autopsies in a large hospital in Zagreb, Croatia: a 10-year retrospective study

**DOI:** 10.3325/cmj.2012.53.48

**Published:** 2012-02

**Authors:** Ivana Pavić, Petra Radulović, Tatjana Bujas, Melita Perić Balja, Jelena Ostojić, Drinko Baličević

**Affiliations:** 1 Ljudevit Jurak University Department of Pathology, Sestre Milosrdnice University Hospital Center, Zagreb, Croatia; 2 Department of Pathology and Forensic Medicine, General Hospital Karlovac, Karlovac, Croatia; 3 Department of Internal Medicine, Division of Clinical Immunology, Pulmonary Diseases and Rheumatology, Sestre Milosrdnice University Hospital Center, Zagreb, Croatia; 4 School of Dentistry, University of Zagreb, Zagreb, Croatia

## Abstract

**Aim:**

To assess the frequency and forms of pulmonary tuberculosis at autopsy in a high-traffic hospital in the capital city of a country with a low tuberculosis incidence.

**Methods:**

We performed a retrospective search of autopsy data from the period 2000 to 2009 at Sestre Milosrdnice University Hospital Center, Zagreb, Croatia. We also examined patients’ records and histological slides.

**Results:**

Of 3479 autopsies, we identified 61 tuberculosis cases, corresponding to a frequency of 1.8%. Active tuberculosis was found in 33 cases (54%), 23 of which (70%) were male. Of the 33 active cases, 25 (76%) were clinically unrecognized and 19 (76%) of these were male.

**Conclusion:**

Clinically undiagnosed tuberculosis accounted for a substantial proportion of active tuberculosis cases diagnosed at autopsy. Autopsy data may be an important complement to epidemiological data on tuberculosis frequency.

Each year, there are nearly 9 million new tuberculosis cases globally and nearly 2 million tuberculosis-related deaths (1,2). Tuberculosis occurs throughout the world, but its incidence varies greatly (3). Preventing infection through contact between healthy individuals and patients is the best measure to fight tuberculosis. The new World Health Organization strategy to fight tuberculosis, Stop TB Strategy (2006-2015), deals with the human immunodeficiency virus epidemic that has increased the incidence of tuberculosis (4). The European Centre for Disease Prevention and Control in 2008 created a strategy against tuberculosis called the “Framework Action Plan to Fight Tuberculosis in the European Union” (5). The long-term goal of the Stop TB Strategy and TB Framework Action Plan is to control and ultimately eliminate tuberculosis in the world based on four basic principles: ensure prompt and quality care for all; strengthen the capacity of health systems; develop new tools; and build partnerships and collaboration with countries and stakeholders (4,5).

Croatia has a low incidence of tuberculosis, which has been steadily decreasing for the last five decades (6). The peak of the epidemic was at the turn of the 19th and 20th century, when more than 400 deaths per 100 000 people occurred as a direct result of tuberculosis (6). In the mid-20th century, the incidence of new tuberculosis cases was 20 000 per 100 000 people (6). In 2009, the incidence of new tuberculosis cases was 20 per 100 000 people (7) and in 2006 nearly all reported cases showed low levels of multidrug resistance (2,6,7). In accordance with international and European efforts, Croatia has its own guidelines for the fight against tuberculosis, with the following goals: to cure at least 85% of cases; to detect at least 70% of tuberculosis patients, and to decrease the incidence of the disease to 10 per 100 000 people (6-8).

Although tuberculosis can affect any organ, 70%-80% of cases suffer from pulmonary tuberculosis (2). Generally, it is possible to detect tuberculosis infection 8-10 weeks after exposure based on a positive tuberculin skin test or an interferon-gamma release assay (9). The rest of the cases have latent tuberculosis infection (LTBI), which is an asymptomatic condition, and cannot transmit the disease (1,2). However, transmission becomes possible under certain conditions such as stress or immune suppression (6,10,11). It is believed that individuals with LTBI account for most infections in low-incidence countries like Croatia, and that this problem is compounded by migration and increasing numbers of homeless persons, alcoholics, and drug addicts (6,10,12).

Statistics about tuberculosis prevalence may underestimate the number of infected people, since as many as half of the cases of pulmonary tuberculosis seen at autopsy were previously undiagnosed (12,13). In fact, few studies have examined the relationship between tuberculosis diagnoses at autopsy and reported tuberculosis prevalence in the population (14). This information may help assess whether clinically unrecognized tuberculosis poses a significant public health threat. The present study examined 3479 autopsies performed from 2000 through 2009, to assess the frequency and forms of pulmonary tuberculosis in a country with a low tuberculosis incidence. The results were compared with the number of tuberculosis patients in Croatia recorded in the Croatian Health Service Yearbook for the same period (7,8).

## Material and methods

The autopsy reports in our database were retrospectively studied for diagnosis of tuberculosis proved by light microscopy. We recorded age, sex, cause of death, and type of tuberculosis diagnosed at autopsy. We also divided autopsy cases into active and inactive tuberculosis and clinically diagnosed and undiagnosed tuberculosis. Confirmative findings of active tuberculosis were hematoxylin and eosin slides with proven granulomatous inflammation with caseous necrosis, and/or positive corresponding Ziehl-Neelsen stains on autopsy material, or culture-positive tuberculosis if it was available (15). The postmortem diagnosis was compared with clinical diagnosis. Inactive tuberculosis was confirmed based on fibrosis and calcification observed at the apex of the lung, clinically and histologically comparing autopsy material with the anamnesis data about previously active tuberculosis (16). Unrecognized tuberculosis was defined as clinically undiagnosed tuberculosis proven on autopsy. Selected results were compared with the epidemiological data from the Croatian Health Service Yearbook for 2000-2009 (7,8).

### Statistics

Hospital tuberculosis proportion was calculated based on the total number of in-hospital deaths per year (range from 1144-1231) and an autopsy rate of about 30% (range 25%-36%) in relation to the corresponding number of autopsies in the same period (range 297-444) (Table 1). For calculation we used Excel Microsoft Office 2003 (Microsoft, Redmond, WA, USA).

**Table 1:** Frequency and characteristics of tuberculosis cases found at autopsy by year

	2000	2001	2002	2003	2004	2005	2006	2007	2008	2009	Total
Total number of in-hospital deaths	1210	1144	1214	1203	1185	1181	1163	1205	1189	1231	11 925
Autopsies (n)	324	297	364	324	327	331	350	444	394	324	3479
Active cases of tuberculosis											
total (n, M/F)	1/1	1/0	6/3	6/3	1/2	2/0	1/0	3/1	1/0	1/0	23/10
median age in years, M/F	80/85	56/-	65/71	66/81	32/75	53/-	40/-	71/70	69/-	55/-	59/76
clinically undiagnosed (n, M/F)	1/1	0/0	6/1	4/3	1/1	2/0	1/0	3/0	1/0	0/0	19/6

*M/F – male/female.

## Results

During the 10-year study period, 61 tuberculosis cases were identified at 3479 autopsies, corresponding to a frequency of 1.8%. The number of all tuberculosis cases (clinically diagnosed and undiagnosed) recognized at autopsy per year was 3-10. Thirty-seven out of all 61 tuberculosis cases diagnosed at autopsy were men (61%) (median 61 years; range 40-92 years) and 24 (39%) were women (median 75 years, range 50-92 years).

Out of all 61 tuberculosis autopsy cases, active tuberculosis was found in 23 men and 10 women (corresponding to 54% of all autopsy active tuberculosis cases). Out of 33 active tuberculosis cases, 25 (76%) were diagnosed only at autopsy. The proportion of clinically unrecognized tuberculosis (19 of 23; 83%) was higher among men than among women (6 of 10; 60%) (Table 1). In 25 (41%) of all 61 tuberculosis cases found at autopsy, death occurred as a direct result of tuberculosis that was not diagnosed and was clinically unrecognized. In these 25 cases, the length of hospitalization was 48 hours or less and none of the available tuberculosis tests was performed. In 8 of 61 (13%) of all autopsy tuberculosis cases, the length of hospitalization was between 3-10 days and acid-fast bacillus smear or culture was performed but death had occurred before the laboratory results were done.

**Figure 1:**
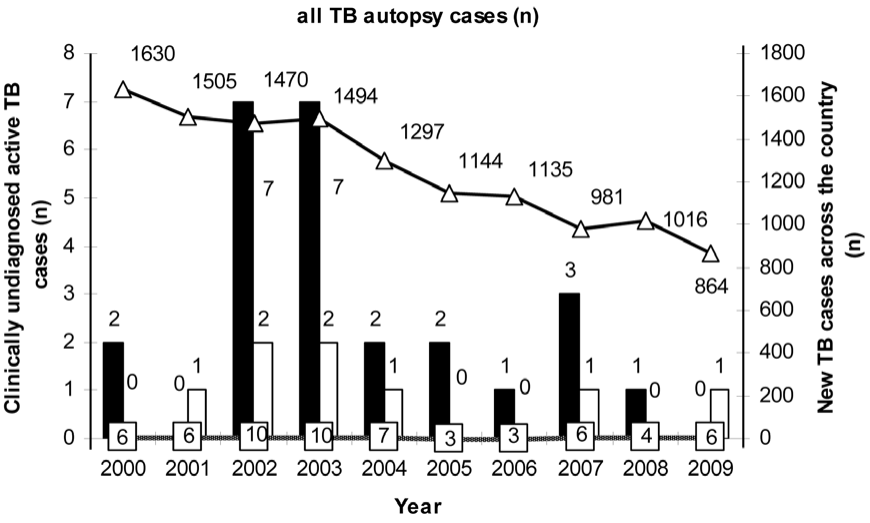
Clinically undiagnosed (black bar) and diagnosed (white bar) active tuberculosis (TB) cases at autopsy in relation to all TB autopsy cases (square) and new TB cases across the country (triangle) (7,8).

We compared our results with the epidemiological data of the Croatian Health Service Yearbook for 2000-2009 (7,8). Clinically undiagnosed tuberculosis (25 of 61 cases) made up a substantial proportion of active tuberculosis cases diagnosed at autopsy, in particular for 2002 and 2003, when 7 active but clinically undiagnosed tuberculosis cases were identified each year (Table 1, Figure 1). These years also saw a slight increase in the number of active tuberculosis cases in the general population although overall disease showed continuous decline in the 10-year period (7,8) (Figure 1).

## Discussion

The present study showed that clinically undiagnosed tuberculosis made up a substantial proportion of active tuberculosis cases diagnosed at autopsy (54%) in a high-traffic city hospital in a country with a low tuberculosis prevalence. Especially indicative were those 41% (25 of 61) of active tuberculosis cases that had not been clinically suspected. These findings showed that autopsy still had value for field-testing epidemiological data, and that pathologists should exercise caution in performing potentially high-risk autopsies. Although tuberculosis has been brought under control in many countries, including Croatia, it continues to persist in the population. In particular, cases of tuberculosis that are clinically undetected undermine the efforts to control the disease and decrease the reliability of epidemiological data.

Our results did not differ from other studies conducted in hospitals of similar size (17-21). Andrion et al (20) and Lum and Koelmeyer (21) found that the frequency of active tuberculosis at autopsy was 1.9%, and that 70% of those cases were diagnosed only at autopsy. Rowinska-Zakrzewska et al (22) found that the proportion of active tuberculosis cases diagnosed only at autopsy was higher in the period 1982-92 (54%) than 1972-81 (24%).

Numerous publications have explained the importance of safe procedures for high-risk autopsies (23-29). Our results and those of others (19-22,30) show that autopsies performed on individuals who died soon after having been admitted to hospital can be a source of highly contagious diseases.

Clinically unrecognized active tuberculosis occurred more often in men than women, especially in younger age groups, as was also reported previously (20,21). This may reflect everyday life habits, especially consumption of tobacco and alcohol, or difficult job conditions such as working outdoors or in poorly ventilated spaces (12,30).

Our results showed that autopsy can be a valuable tool for epidemiological monitoring. However, Croatian law allows families to refuse it, especially in cases when the deceased was hospitalized for more than one day (31). Frequently, the deceased are the elderly with pneumonia who did not adequately respond to treatment with antibiotics and presented with dyspnea, non-specific symptoms, and atypical radiographic appearance (27-30,32,33). Our results suggest that performing autopsies on these and other cases can alert health care workers to “invisible infections” that can occur in families without diagnosed tuberculosis.

This study represents a valuable contribution to the epidemiological studies of tuberculosis, but the results should be interpreted with caution. The greatest limitation of our study is that we were not able to determine the exact number of in-hospital tuberculosis deaths (active and inactive forms). This was impossible to avoid since not every in-hospital death was subject to autopsy, especially in case of old people with many comorbidites and chronic diseases where tuberculosis was often not included in differential diagnosis (31,34).

Clinically unrecognized tuberculosis, not proven at autopsy, becomes an invisible form of infection. Autopsy findings can help in cases when monitoring, prevention, and drug treatment cannot prevent the infection of healthy population, including families and medical personnel. This particularly applies to the periods when active clinically unrecognized tuberculosis is on the increase. Continued morphological analysis at autopsy can be a source of valuable information on regional and global dynamics of pulmonary tuberculosis and on the quality of epidemiological monitoring of disease trends.
